# Reinitiation of antidepressant pharmacotherapy among patients discharged from the hospital: A population-based cohort study

**DOI:** 10.1371/journal.pmen.0000427

**Published:** 2026-03-25

**Authors:** Waseem Abu-Ashour, John-Michael Gamble, John Hawboldt, Joanna E. M. Sale

**Affiliations:** 1 School of Pharmacy, Memorial University, St. John’s, Newfoundland and Labrador, Canada; 2 School of Pharmacy, University of Waterloo, Kitchener, Ontario, Canada; 3 Li Ka Shing Knowledge Institute, St. Michael’s Hospital, Unity Health Toronto, Toronto, Ontario, Canada; 4 Institute of Health Policy, Management & Evaluation, University of Toronto, Toronto, Ontario, Canada; 5 Department of Surgery, Faculty of Medicine, University of Toronto, Toronto, Ontario, Canada; West China Hospital of Sichuan University, CHINA

## Abstract

To examine the incidence of antidepressant medication reinitiation following a ≥ 6-month gap as a proxy for relapse in patients with major depressive disorder (MDD) discharged from hospital and followed in primary care, and to assess patterns of antidepressant use before and after reinitiation, as well as associations with socio-demographic and clinical variables. We conducted a population-based cohort study using seven linked administrative health databases from Newfoundland and Labrador, Canada. Adults (≥18 years) with a first hospitalization for MDD between June 2017 and March 2023 and a post-discharge antidepressant prescription of ≥30 days were included. The primary outcome was reinitiation of antidepressant pharmacotherapy following a ≥ 6-month treatment gap. Antidepressant treatment groups were defined as SSRI monotherapy (reference), SNRI monotherapy, other monotherapy, two-medication combination therapy, and three or more medication combinations. Time-varying Cox regression models were used to assess associations with reinitiation risk, adjusted for age, sex, socioeconomic status (SES), and length of hospital stay. Sensitivity and exploratory age-stratified analyses were conducted. Among 2,734 patients, 61% reinitiated antidepressant treatment after a ≥ 6-month gap. SSRI monotherapy was the most common initial regimen (34.3%), followed by 2-medication combinations (18.1%). Combination therapy was associated with lower reinitiation risk compared to SSRIs: HR = 0.67 (95% CI: 0.50–0.90) for 2-medication combinations and HR = 0.49 (95% CI: 0.40–0.60) for 3 + medication combinations. SNRI monotherapy conferred modest protection (HR = 0.84, 95% CI: 0.72–0.99). Age, sex, and SES were independently associated with reinitiation. Younger adults, males, and individuals in both high and low-income quintiles were at increased risk. Reinitiation of antidepressants after a ≥ 6-month gap was common following hospitalization for MDD. Combination therapy maybe associated with reduced reinitiation risk compared to SSRI monotherapy, with age-specific treatment effects, although residual confounding cannot be excluded. Further research, ideally RCTs, is needed before informing clinical decision-making.

## Introduction

Major depressive disorder (MDD) is one of the most prevalent mental disorders and the leading cause of disability worldwide [1]. The Global Burden of Diseases, Injuries, and Risk Factors Study (GBD) 2019 showed that MDD was one of the two most disabling mental disorders ranked among the top 25 leading causes of burden worldwide in 2019 [2,3]. Although acute phase treatment has been well established and effectively improves the symptoms of MDD, the risk of relapse remains a significant problem globally [4]. The prevention of relapse in MDD one of the most important challenges to its management [5]. Relapse refers to a clinically significant return of depressive symptoms that again meet full syndromal criteria for a MDD episode, occurring before a patient has achieved recovery [6]. Risk factors include the presence of residual depressive symptoms—defined in the literature as persistent subsyndromal symptoms that remain after acute-phase treatment but fall short of full syndromal criteria for a major depressive episode—along with a prior history of relapse, comorbid anxiety disorders, and childhood maltreatment [7], as well as the severity of the MDD episodes [8,9]. Buckman et al. note that primary studies vary in their operational thresholds for ‘residual symptoms,’ but consistently identify this broad category of post-treatment symptom persistence as a strong prognostic indicator of subsequent relapse or recurrence. There are multiple indicators of a MDD relapse in claims data, such as suicide attempts, psychiatric hospitalizations, electroconvulsive therapy, and reinitiation of antidepressant pharmacotherapy after a ≥ 6-month gap [10,11].

Despite primary care being the most common point of healthcare contact for patients with MDD, research in this setting remains limited [12,13]. Studies targeting MDD in primary care focus primarily on newly diagnosed MDD cases rather than relapse prevention [14] or have only evaluated the efficacy of specific interventions on relapse [15–20]. A systematic review and meta-analysis of observational studies on MDD in primary care emphasized the necessity of larger, long-term follow-up cohorts to establish reliable relapse estimates [21]. Moreover, there is a need for additional studies to enhance our understanding of MDD relapse-associated risk factors in primary care [13].

Therefore, we sought to conduct a cohort study to measure the incidence of antidepressant medication reinitiation after a ≥ 6-month gap as an indicator of MDD relapse. We also describe antidepressant medication use before and after the time of antidepressant reinitiation and measure the association between age, sex, and socioeconomic status and antidepressant medication reinitiation.

## Methods

### Study design and data sources

A cohort study using seven databases was conducted. The custodian of these databases were the Newfoundland and Labrador Centre for Health Information (NLCHI). NLCHI is responsible for developing and implementing the province of Newfoundland and Labrador confidential and secure electronic health record. The study was approved by the Newfoundland and Labrador Health Ethics Research Board (# 20240277). Reporting followed the STROBE guidelines (S1 Checklist). Data linkage was performed deterministically by NLCHI using their internal de-identification and using the unique provincial Medical Care Plan (MCP) number, which serves as the master person identifier across all clinical information systems. Multiple MCP numbers belonging to the same individual are collapsed into a single internal identifier, which is then converted into a study ID for the released dataset. This approach minimizes false matches and mitigates missed matches typically associated with deterministic linkage. All datasets were subsequently de-identified by NLCHI to ensure patient confidentiality, and researchers accessed the anonymized datasets through the secure NLCHI Data Lab virtual environment from 08/09/2023–01/06/2024 (S1 Table):

### Provincial discharge abstracts database

Demographic, clinical and administrative data for inpatient hospitalizations and surgical daycare procedures occurring at acute care facilities in the province.

### Client registry

Demographic and administrative information—including year and month of birth, sex, and census sub-division—enables accurate identification of individuals in the provincial electronic health record. Deterministic linkage was performed using the unique provincial MCP number. These systems map to the MCP-based index to ensure consistent linkage across datasets. For privacy protection, NLCHI encrypts the MCP number before releasing data to the research team.

### MCP fee-for-service physician claims database

Information related to services provided by fee-for-service physicians; includes patient demographics, provider’s information, and information on the encounter.

### NLCHI mortality system

Demographic, administrative and clinical data related to all deaths that occur in the province; includes health conditions present at time of death; also includes underlying cause of death, as determined by Statistics Canada.

### The pharmacy network

Demographic, medication, and clinical information for individuals accessing community or hospital outpatient pharmacies in the province, including details on prescriptions, over-the-counter products, and allergies.

### Statistics Canada census

Data on the province residents includes demographic, social and economic characteristics such as age, sex, marital status, family size, income, education, ethnicity, language, and citizenship and immigration.

### Postal code conversion file

The Postal Code Conversion File [22] links six-character postal codes to standard geographic areas such as dissemination areas, census tracts, and census subdivisions. NLCHI uses Statistics Canada postal code linkage procedures; however, as with other administrative datasets, no formal local evaluation of postal code linkage error is available.

### Study population

The study period was June 1, 2017, to March 1, 2023. Individuals were followed from the time they entered the cohort until either the end of the study period, death, or they were lost to follow-up. In this cause-specific hazard framework, death was treated as a censoring event, consistent with approaches used to estimate relative hazards. The inpatient sample in this study reflects local healthcare capacity. Access to hospital admission may not mirror hospitalization thresholds in health systems with more constrained psychiatric resources.

### Inclusion

Our cohort included patients aged ≥18 with a first hospitalization for MDD between June 1, 2017, and March 1, 2023. Hospitalizations for MDD were identified using ICD-10 diagnosis codes, with a gap of at least one year to a previous MDD diagnosis to identify newly observed hospitalizations for MDD in this dataset [23,24]. Individuals who had a previous hospitalization for MDD or a prescription record for an antidepressant within the 12 months prior to their admission date were excluded. Evidence has shown that remission of MDD occurs within 3 months to 1 year [24]. The definition of MDD included patients with adjustment disorder, dysthymic disorder, depression not otherwise specified, and history of one or more depressive episodes to capture all potential MDD patients, including those who may not have received a properly coded MDD diagnosis [25]. This was done to ensure the capture of all MDD cases within the database, where MDD is sometimes undercoded or recorded nonspecifically in administrative databases. To be included in this incident study cohort, patients must also have received at least one prescription for an antidepressant medication. Additionally, the minimum window for inclusion required at least a 30-day prescription period. This was to ensure that only prescriptions reflecting sustained treatment were included in the analysis.

### Exclusion criteria

Patients who were pregnant and those with a diagnosis of personality disorders, schizophrenia or bipolar disease before index date were excluded. Most studies assessing the impact of comorbid psychiatric disorders on antidepressant response rates have found that the presence of a comorbid personality disorders, schizophrenia or bipolar disease, conveys a worse prognosis [26–29]. Patients were also excluded if they had incomplete prescription information (name, dose, quantity, duration), or prescriptions with a duration of less than 30 days.

### Exposure groups

Antidepressant medications were grouped into 5 groups; selective serotonin reuptake inhibitors (SSRI) monotherapy; serotonin and norepinephrine reuptake inhibitors (SNRI) monotherapy; other monotherapy (other antidepressant monotherapy); combination therapy (two antidepressant medications); and combination therapy (three or more antidepressant medications). All exposure variables were derived from dispensing records in the Pharmacy Network. Antidepressant class and regimen were determined using drug identification numbers and overlapping days’ supply. Because all dispensing is captured through the same centralized system, assessment methods were consistent across all treatment groups.

### Outcomes

The primary outcome in this study was the incidence of reinitiation of antidepressant medication after a ≥ 6-month gap following the previous prescription in adults ≥18 years old with an MDD diagnosis identified in hospital and followed up in primary care who are new users of an antidepressant. Type of antidepressant use within the MDD cohort and time on treatment was determined.

### Covariates

Data on socio-demographic and clinical variables that could increase the incidence of MDD relapse in adults with MDD were collected. These covariates were chosen based on the literature [30,31] as well as data availability from the datasets included. Variables collected included age at index date, sex (Male, Female), socioeconomic status (Quantile 1 [<$25,000], Quantile 2 [$25,000 – $34,999], Quantile 3 [$35,000 – $44,999], Quantile 4 [$45,000 – $54,999], Quantile 5 [>-$55,000]) and length of hospital stay (1 week, 30 days, 60 days, ≥ 120 days). Using census data, median income in each dissemination area was calculated and each neighborhood was divided into income quintiles, with quintiles 1 and 5 having the lowest and highest median incomes, respectively [25,32]. Other clinically relevant confounders—such as baseline severity of MDD, psychotherapy use, substance use, and lifetime psychiatric history—were not available in administrative data and could not be included.

### Statistical analysis

Descriptive statistics were conducted to compare the baseline demographic, clinical and treatment characteristics for the study cohort. Categorical variables were reported as a frequency and percentage, and continuous variables were reported as means (standard deviation [SD]) or medians (interquartile range).

The primary analysis examined reinitiation of antidepressant medication risk across the five treatment groups for the full follow-up period. Incidence rates of reinitiation of antidepressant medication were calculated per 1000 person-years with 95% confidence intervals for each treatment group. This approach provided a standardized measure of reinitiation of antidepressant medication frequency that accounted for varying follow-up times across treatment groups, allowing for direct comparison of reinitiation of antidepressant medication risk between different medication groups. Kaplan-Meier curves were generated to visualize cumulative incidence of reinitiation of antidepressant medication for the overall population, providing intuitive representation of temporal patterns in reinitiation of antidepressant medication risk. The number of patients at risk of reinitiation of antidepressant medication was reported at regular time intervals to indicate the precision of estimates over time.

Time-varying Cox regression models were conducted to account for dynamic changes in medication exposure throughout the follow-up period. SSRI monotherapy was selected as the reference group because they are generally recommended as first-line pharmacotherapy treatment options [33,34], and they were the most commonly prescribed type of antidepressant [35]. Using SSRIs as the reference group allows for meaningful comparisons with other treatment patterns to assess reinitiation of antidepressant medication risks. A time-varying approach was chosen over traditional cox regression to avoid immortal time bias and to accurately capture the temporal relationship between changing medication patterns and outcomes [36–40]. Potential confounding variables included in the model were identified based on biological rationale, clinical experience, data availability, and established associations reported in prior studies on MDD relapse. Specifically, the model adjusted for age, sex, socioeconomic status, and length of hospital stay [5,41–45]. Incidence rates per 1,000 person-years are presented to provide unadjusted summary measures of event frequency across treatment groups. Adjusted hazard ratios were prioritized as the primary comparative measures because they account for key demographic and clinical confounders known to influence relapse risk.

Sensitivity analyses were conducted which included time-varying cox regression for 365-day and 730-day follow-up periods to assess both short and medium-term treatment effects, reflecting clinically relevant timeframes for reinitiation of antidepressant medication prevention. Moreover, a fixed exposure cox regression analysis was also conducted to complement the time-varying results [36–40]. Due to clinical interest in age-related differences in antidepressant treatment response [46], we conducted exploratory stratified analyses by age group. The cohort was divided into three age categories: 18–40, 41–65, and >65 years. Time-varying Cox regression models were applied separately within each stratum to examine patterns of association between treatment type and reinitiation risk. These analyses were intended to describe age-specific trends and were designed to formally test for statistical interaction.

Several steps were taken to reduce potential sources of bias inherent in observational administrative data research. To minimize selection bias, we used population-based administrative datasets with province-wide capture of hospitalizations, physician services, pharmacy dispensing, and mortality, ensuring inclusion of eligible individuals. Misclassification bias was reduced by relying on standardized ICD-10-CA diagnostic codes assigned by trained health information professionals, and by using pharmacy dispensing records that capture all outpatient prescriptions processed through the provincial Pharmacy Network. Time-varying Cox regression was used to reduce immortal time bias and to more accurately reflect dynamic changes in antidepressant exposure over time, thereby minimizing exposure misclassification. Immortal time bias occurs when periods during which the outcome cannot occur (e.g., the time before initiating or switching an antidepressant) are misclassified as exposed time, artificially lowering risk estimates. In our model, each patient’s exposure category was updated whenever their antidepressant regimen changed, ensuring that person-time was attributed only to the treatment actually received at each point in time. This approach appropriately accounts for dynamic exposure patterns. Confounding was addressed by including the available covariates in all multivariable Cox regression models. Missing data were minimal and occurred primarily in the SES variable. Consistent with best practices in administrative database research, individuals with missing SES information were retained in the analysis by including a separate “missing” category to avoid potential selection bias. No other variables had missingness requiring imputation. Participants contributed person-time from cohort entry until reinitiation, death, or end of follow-up. Loss to follow-up was handled by censoring individuals at their last known date in the dataset under the assumption of non-informative censoring in cox models. Deaths were treated as censoring events because cause-specific relapse information was not available; therefore, competing risks could not be modeled directly.

A priori sample size calculations indicated that 296 participants were required to detect a 20% relative difference in relapse risk with 80% power. Because this was a population-based administrative cohort, all eligible individuals during the study period were included, resulting in a final sample of 2,841 patients. All analyses were conducted using R, and statistical tests were 2-sided with a significance threshold of p < 0.05.

## Results

A total of 2,841 patients were admitted to the hospital with MDD, between 2017 and 2023 were included in the study, with an average follow-up period of 2.4 years. The mean age of the cohort was 55.2 years, with a slightly higher proportion of female patients (56%). Socioeconomic distribution showed predominance in middle-income categories. The demographic and clinical characteristics of the study population are detailed in Table 1.

**Table 1:** Characteristics for 2841 Patients Hospitalized with a Diagnosis of Major Depressive Disorder between 2017 and 2022, Newfoundland and Labrador, Canada.

Variable	n (%)
**Total Number of study participants**	2841
**Age at cohort entry**	
Mean (SD)	55.2 (19.8)
Groups n (%):	
18-30 years	398 (14.0%)
31-40 years	392 (13.8%)
41-50 years	366 (12.9%)
51-65 years	705 (24.8%)
>65 years	980 (34.5%)
**Sex, n (%):**	
Female	1588 (55.9%)
Male	1253 (44.1%)
**Social Economic Status (SES), n (%):**	
Quantile 1 (<$25,000)	28 (1.09%)
Quantile 2 ($25,000 – $34,999)	969 (34.1%)
Quantile 3 ($35,000 – $44,999)	1407 (49.5%)
Quantile 4 ($45,000 – $54,999)	181 (6.4%)
Quantile 5 (>-$55,000)	130 (4.6%)
NA*	126 (4.4%)
**Length of Initial Hospitalization:**	
**Total (mean, SD)**	18.1 (32.1)
Groups:	
1 week (%)	35.82%
30 days (%)	30.30%
60 days (%)	10.10%
≥120 days (%)	6.76%

*NA: Missing SES due to non-geocodable postal codes from StatCanada; these patients were retained as a separate category in the regression models.

**Fig 1:**
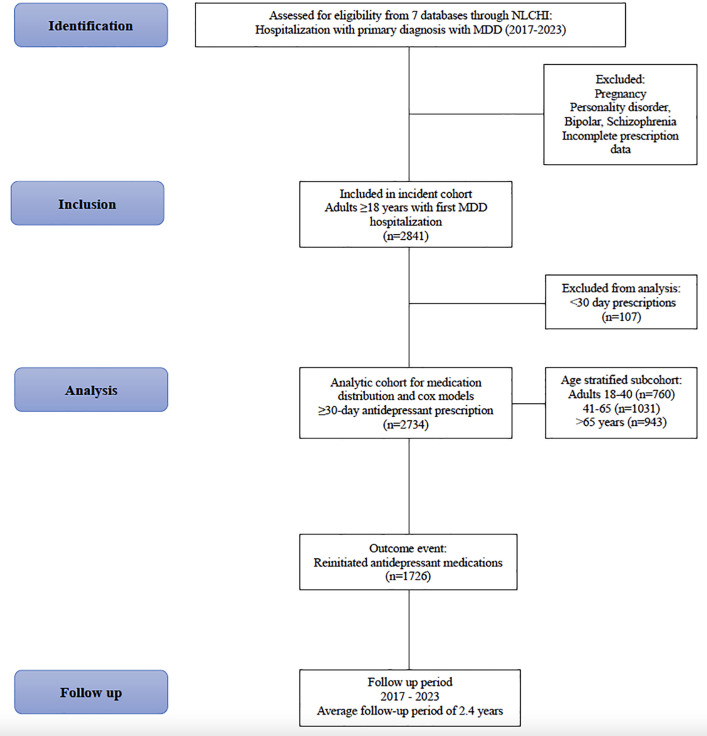
Flowchart of Cohort Selection.

In the initial cohort of 2,841 hospitalized patients, 2,734 individuals filled an outpatient antidepressant prescription lasting ≥30 days. These 2,734 patients comprised the analytic cohort for the medication distribution analysis and all Cox regression models. The remaining 107 patients filled prescriptions lasting <30 days (Fig 1). Table 1 reflects the full NLCHI cohort (n = 2,841), whereas Table 2 and all regression analyses use the analytic cohort of 2,734 patients. SSRI monotherapy was the most common initial treatment post-hospital discharge (34.3%), followed by 2-medication combination therapy (18.1%) and other monotherapy (16.5%). There were 1,726 patients (61%) who reinitiated an antidepressant after a 6-month gap (Table 2). At the time of discontinuation preceding reinitiation, SSRI monotherapy remained the predominant treatment (43.3%), with SNRI monotherapy (17.2%) and other monotherapy (20.3%) also commonly used (Table 2). However, following reinitiation, there was a dramatic shift toward combination therapies, with only 29.6% remaining on any form of monotherapy while 62.3% received combination regimens (29.9% on 2-medication combinations and 32.4% on 3 + medication combinations).

**Table 2:** Study Cohort Medication Distribution.

Medication	Medication Post Hospital Discharge (2734 patients)	Medication At Time of Reinitiation of Antidepressant Medication (1726 patients)	First Medication Post Reinitiation of Antidepressant Medication (1726 patients)
**SSRI Monotherapy:**	**937 (34.3%)**	**747 (43.3%)**	**270 (15.6%)**
Citalopram	231 (24.7%)	160 (21.4%)	57 (21.1%)
Escitalopram	138 (14.7%)	121 (16.2%)	41 (15.2%)
Fluoxetine	99 (10.6%)	97 (13.0%)	33 (12.2%)
Fluvoxamine	6 (0.6%)	9 (1.2%)	s
Paroxetine	94 (10.0%)	104 (13.9%)	34 (12.6%)
Sertraline	369 (39.4%)	256 (34.3%)	103 (38.1%)
**SNRI Monotherapy:**	**363 (13.3%)**	**298 (17.2%)**	**113 (6.5%)**
Desvenlafaxine	s	8 (2.7%)	s
Duloxetine	37 (10.2%)	41 (13.7%)	12 (10.6%)
Venlafaxine	321 (88.4%)	249 (83.6%)	100 (88.5%)
Levomilnacipran	s	0	0
**Other Monotherapy:**	**452 (16.5%)**	**351 (20.3%)**	**130 (7.5%)**
Bupropion	83 (18.4%)	61 (17.4%)	24 (18.5%)
Mirtazapine	247 (54.6%)	115 (32.8%)	51 (39.2%)
Moclobemide	0	s	s
Phenelzine	0	s	0
Tranylcypromine	0	0	s
Trazadone	46 (10.2%)	59 (16.8%)	18 (13.8%)
Tryptophan	0	s	0
Vilazodone	0	s	s
Vortioxetine	6 (1.3%)	13 (3.7%)	s
Amitriptyline	33 (7.3%)	57 (16.2%)	11 (8.5%)
Clomipramine	15 (3.3%)	11 (3.1%)	5 (3.8%)
Desipramine	5 (1.1%)	6 (1.7%)	6 (4.6%)
Doxepine	0	s	0
Imipramine	0	s	0
Nortriptyline	16 (3.6%)	21 (6.0%)	10 (7.7%)
Trimipramine	s	s	0
**2 Combination Therapy**	**495 (18.1%)**	**121 (7.0%)**	**516 (29.9%)**
**3 + more Combination Therapy**	**98 (3.6%)**	**209 (12.1%)**	**559 (32.4%)**
No Record*	**389 (14.2%)**	**0**	**138 (8.0%)**

*No Record: there was no record of these patients that took any medications – could be due to loss of follow up, move outside of the province or missing.

**s:** Small cell counts suppressed (≤5) to minimize the risk of deductive disclosure, consistent with common practices in administrative health data reporting.

**Patients excluded from medication groups:** Population total is 2841. Patients on medications included was 2734. The difference of 107 patients had medications <30 days prescription and were excluded in this medication distribution, as the time period window for inclusion for follow up was at least 30 days duration.

**Note:** Counts in each medication category reflect the number of dispensed antidepressant records during the follow-up period, not the number of unique patients because patients often experienced multiple treatment changes over time. Accordingly, counts across categories should not be expected to sum to the total cohort size.

**Fig 2:**
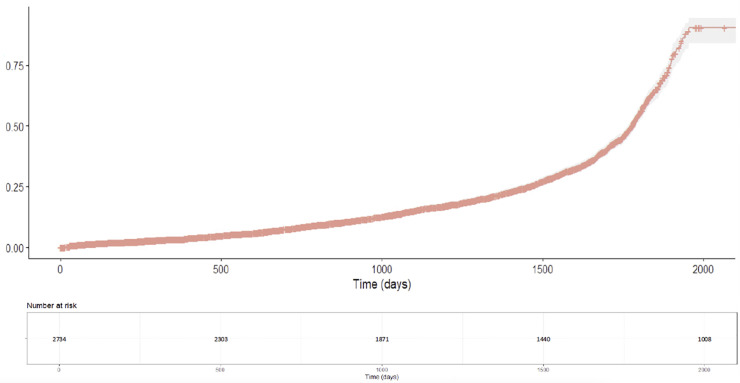
Cumulative Incidence of Reinitiation of Antidepressant Medication for Entire Population.

The Kaplan-Meier cumulative incidence analysis for the entire study population showed an association with a progressive increase in reinitiation of antidepressant medication risk over time, with three distinct phases (Fig 2).

Combination therapy with 2 medications was associated with the lowest reinitiation of antidepressant medication rate (94 per 1000 person-years), followed by combination therapy with 3 + medications (89 per 1000 person-years). SNRI was associated with an intermediate rate (161 per 1000 person-years), while SSRI and other monotherapy were associated with similar rates (198 and 182 per 1000 person-years, respectively) (Table 3-A).

**Table 3:** Cox Regression Analysis for Reinitiation of Antidepressant Medication After A Gap Of ≥ 6 Months: A. Main analysis: Time Varying Cox Regression Analysis for Full follow up period. B(1,2,3). Sensitivity Analysis: 1.Time Varying Cox Regression Analysis for 365 days, 2.Time Varying Cox Regression Analysis for 730 days, 3.Fixed Cox Regression Analysis Full Follow Up Period.

A. Full Follow Up					
Medication Group	Number of events of reinitiating an antidepressant	Incidence Rate (per 1000 person-years)	HR	95% CI	P value
SSRI	700	198	Ref	Ref	Ref
SNRI	307	161	0.84	0.72 - 0.99	P = 0.032
Monotherapy	497	182	1.09	0.95 - 1.25	P = 0.210
Combination Therapy (2 medications)	71	94	0.67	0.50 - 0.90	P = 0.007
Combination Therapy (3 + medications)	151	89	0.49	0.40 - 0.60	P < 0.001
**B.(1) 365 Days Follow up**					
**Medication Group**	**Number of events of reinitiating an antidepressant**	**Incidence Rate (per 1000 person-years)**	**HR**	**95% CI**	**P value**
SSRI	128	178	Ref	Ref	Ref
SNRI	56	119	0.78	0.67 - 0.91	P = 0.002
Monotherapy	91	154	0.93	0.81 - 1.06	P = 0.284
Combination Therapy (2 medications)	13	63	0.40	0.30 - 0.53	P < 0.001
Combination Therapy (3 + medications)	28	79	0.56	0.46 - 0.69	P < 0.001
**B.(2) 730 days follow up**					
**Medication Group**	**Number of events of reinitiating an antidepressant**	**Incidence Rate (per 1000 person-years)**	**HR**	**95% CI**	**P value**
SSRI	256	196	Ref	Ref	Ref
SNRI	112	161	0.81	0.70 - 0.95	P = 0.0102
Monotherapy	181	178	0.99	0.87 - 1.13	P = 0.9032
Combination Therapy (2 medications)	26	71	0.48	0.36 - 0.65	P < 0.001
Combination Therapy (3 + medications)	55	97	0.55	0.45 - 0.68	P < 0.001
**B.(3) Fixed Cox Regression Analysis Full Follow Up Period**					
**Medication Group**	**Number of events of reinitiating an antidepressant**	**Incidence Rate (per 1000 person-years)**	**HR**	**95% CI**	**P value**
SSRI	863	224	Ref	Ref	Ref
SNRI	290	174	0.68	0.57 - 0.80	P < 0.001
Monotherapy	376	197	0.88	0.76 - 1.03	P = 0.110
Combination Therapy (2 medications)	83	109	1.01	0.76 - 1.34	P = 0.961
Combination Therapy (3 + medications)	114	92	0.28	0.22 - 0.36	P < 0.001

Time-varying Cox regression analysis demonstrated associations with reinitiation of antidepressant medication risks across treatment groups during the full follow-up period (Table 3-A). Combination therapy with 3 + medications was associated with the strongest protective effect against reinitiation of antidepressant medication compared to SSRI monotherapy with a 51.1% risk reduction (HR = 0.49, 95%CI 0.40-0.60, p < 0.001). Combination therapy with 2 medications was associated with a 32.7% lower reinitiation of antidepressant medication risk (HR = 0.67, 95% CI: 0.50-0.90, p = 0.007), while SNRI monotherapy was associated with a 15.8% reduction (HR = 0.84, 95% CI: 0.72-0.99, p = 0.032). Other monotherapy showed no significant association with reinitiation of antidepressant medication risk compared to SSRI (HR = 1.09, 95% CI: 0.95-1.25, p = 0.210).

Sensitivity analyses of the 365 day and 730 day follow-up times were consistent with the primary analysis with combination therapy showing superior protective effects against reinitiation of antidepressant medication compared to SSRI (Table 3 B.(1,2)). Fixed exposure analysis over the full follow-up period corroborated the findings related to combination therapy of 3 + medications (Table 3.B(3)).

Exploratory stratified analyses by age group revealed differences in the magnitude and direction of association between medication and antidepressant reinitiation. In patients aged 18–40 years, combination therapy was associated with the strongest protective effects against reinitiation of antidepressant medication. Combination therapy of 2 medications was associated with the lowest reinitiation of antidepressant medication risk (HR = 0.47, 95% CI: 0.28-0.78, p = 0.004), (Table 4-A). For patients aged 41–65 years, only combination therapy of 3 + medications was associated with significant protection (HR = 0.55, 95% CI: 0.40-0.76, p < 0.001). Other treatment strategies, including SNRI monotherapy, other monotherapy, and combination therapy of 2 medications, did not show significant associations with reduced relapse risk compared to SSRI (Table 4-B). In individuals >65 years, combination therapy of 3 + medications was associated with the strongest protection (HR = 0.42, 95% CI: 0.25-0.69, p < 0.001), representing a 58% risk reduction. Neither combination therapy of 2 medications, SNRI, nor other monotherapy showed significant associations with reduced relapse risk compared to SSRI (Table 4-C).

**Table 4:** (A-C). Exploratory Stratified Analyses by Age Group: Time Varying Cox Regression Analysis for Reinitiation of Antidepressant Medication After a Gap Of ≥ 6 Months Based on Age.

A. Group 18–40 Years			
Medication Group	HR	95% CI	P value
SSRI	Ref	Ref	
SNRI	0.88	0.68 - 1.13	P = 0.312
Monotherapy	1.28	1.03 - 1.57	P = 0.023
Combination Therapy (2 medications)	0.47	0.28 - 0.78	P = 0.004
Combination Therapy (3 + medications)	0.56	0.39 - 0.79	P < 0.001
**B. Group 41–65 Years**			
**Medication Group**	**HR**	**95% CI**	**P value**
SSRI	Ref	Ref	
SNRI	0.89	0.69 - 1.14	P = 0.357
Monotherapy	1.12	0.89 - 1.40	P = 0.346
Combination Therapy (2 medications)	1.05	0.69 - 1.60	P = 0.816
Combination Therapy (3 + medications)	0.55	0.40 - 0.76	P < 0.001
**C. Group >65 Years**			
**Medication Group**	**HR**	**95% CI**	**P value**
SSRI	Ref	Ref	
SNRI	0.99	0.67 - 1.46	P = 0.962
Monotherapy	1.04	0.76 - 1.43	P = 0.785
Combination Therapy (2 medications)	0.63	0.33 - 1.23	P = 0.175
Combination Therapy (3 + medications)	0.42	0.25 - 0.69	P < 0.001

Variables associated with reinitiation of antidepressant medication for 1 year follow up were also examined. Age was associated with a protective effect, with each 10-year increase associated with a 3.9% reduction in reinitiation of antidepressant medication risk (HR = 0.961, 95% CI: 0.961-0.961, p < 0.0001). Males were associated with higher reinitiation of antidepressant medication risk compared to females (HR = 1.54, 95% CI: 1.53-1.56, p < 0.0001). Socioeconomic disparities were also associated with reinitiation of antidepressant medication risk, with individuals in the second quintile (HR = 1.29, 95% CI: 1.24–1.33, p < 0.0001) and the highest quintile (HR = 1.33, 95% CI: 1.21–1.45, p = 0.005) showing an increased association with reinitiation of antidepressant medication compared to those in the lowest income groups. Length of hospital stay did not show any association (HR = 1.01, 95% CI: 0.98 - 1.02, p = 0.34).

## Discussion

This cohort study utilized administrative databases from the Province of Newfoundland and Labrador to examine the incidence of antidepressant reinitiation among patients discharged from the hospital due to MDD. Our findings reveal several important patterns in the treatment and outcomes in a cohort of MDD patients discharged from the hospital and followed up in primary care. Approximately two-thirds of patients reinitiated antidepressant medication after a ≥ 6-month gap, indicating a substantial burden of relapse. Combination antidepressant therapy was consistently associated with lower risk of reinitiation relative to SSRI monotherapy. Moreover, age, sex, and SES were also independently associated with reinitiation risk, with younger patients, males, and patients in both low- and high-income groups showing greater vulnerability.

Combination therapy demonstrated the most substantial protective association against antidepressant reinitiation compared to SSRI monotherapy, while SNRI monotherapy to a lesser extent. Overall, these findings suggest patterns of association in which SSRI and other monotherapy regimens were more frequently associated with reinitiation rates, while combination therapies were associated with lower reinitiation rates. These associations should be interpreted cautiously, as treatment intensification may reflect underlying clinical complexity rather than pharmacological benefit. The observed associations of combination therapy align with previous evidence supporting its efficacy in MDD relapse [47–50]. Sensitivity analyses showed broadly similar associations across follow-up periods. At one year, the protective effects of combination therapy were most pronounced, with a 60% risk reduction observed for 2-medication combinations and a 44% reduction for combinations of 3 + medications. These associations persisted at 730 days, suggesting sustained benefit of combination therapy over time. The consistency across time-varying and fixed exposure models further strengthens the inference that more intensive pharmacological strategies may mitigate relapse risk in this population [47–50]. It is important to note that combination therapy might be initiated in clinical practice for patients with partial response, complex symptom profiles, or multiple comorbidities and or patient preference. Without detailed clinical information on treatment rationale or the characteristics of patients selected, we cannot fully determine the reason for the lower reinitiation rates associated with combination strategies.

Age-stratified results suggested differences in treatment-associated outcomes across age. In younger adults (18–40 years), the strongest association with lower reinitiation risk was observed with 2-medication combination therapy. Among older adults, only regimens involving 3 or more medications were associated with reduced risk. While these patterns may reflect underlying differences in illness duration, treatment history, or pharmacodynamics by age [51,52], the stratified analyses were descriptive and not designed to test for treatment by age interaction. As such, these observed differences should not be interpreted as evidence of treatment effect heterogeneity. Age was also inversely associated with reinitiation risk, consistent with prior literature suggesting younger age at onset may predict higher relapse risk [53–55]. However, some studies finding no association between current age and relapse [7]. Our findings suggest that while younger age may be a proxy for factors such as earlier onset, higher symptom burden, or lower adherence, it warrants consideration in clinical decision-making. Male sex was associated with a significantly higher risk of reinitiation, a finding that contrasts with some prior reports suggesting elevated relapse risk among women, particularly following antidepressant discontinuation [56]. However, most systematic reviews indicate that sex alone is not a strong independent predictor of relapse [7,53,57–59]. The elevated risk among men in our cohort may reflect differences in treatment adherence, symptom reporting, or comorbidities that were not fully captured in administrative data. SES was also independently associated with reinitiation risk. Both the second income quintile and the highest quintile had higher risks compared to the lowest, suggesting a non-linear relationship. While some literature finds no consistent association between SES and MDD relapse [7,53], other work has proposed that indirect pathways, such as differential access to care or psychosocial stress exposure, may contribute to risk. Evidence from Canada demonstrates that higher-income groups have disproportionately greater access to psychiatric services despite universal coverage [60], while socioeconomic gradients in mental health need and service use remain well-documented [61]. In contrast, clinical predictors such as prior episodes or residual symptoms—rather than SES—tend to drive recurrence risk in longitudinal studies [7,53]. Our findings may reflect the intersection of these structural and clinical mechanisms, underscoring the need to consider both relative socioeconomic advantage and disadvantage in relapse-prevention strategies. Overall, these findings highlight the chronic and recurrent nature of MDD, with the risk of antidepressant medication reinitiation accumulating over time. While our study specifically measured medication reinitiation after a ≥ 6-month gap as a proxy for relapse, this outcome represents a clinically significant component of the broader relapse phenomenon. The substantial long-term incidence of medication reinitiation emphasizes the critical need for sustained treatment and long-term management strategies to mitigate the MDD relapse in clinical practice.

Several limitations warrant consideration in interpreting our findings. Although deterministic linkage can sometimes increase the risk of missed matches, NLCHI’s internal de-identification and MCP consolidation process is specifically designed to prevent this by ensuring that all MCPs associated with an individual are correctly unified before linkage. As with any linked administrative dataset, a small possibility of linkage error remains, but the risk is substantially minimized in this setting. In addition, deterministic linkage of postal codes may result in higher missed-match rates among individuals with unstable housing, high mobility, or incomplete demographic records. These populations may therefore be under-represented in our cohort, potentially biasing estimates toward healthier and more socioeconomically stable groups.

Our definition of new MDD hospitalization is based on a one-year clean period and captures newly observed episodes rather than true first-ever MDD. Administrative data do not include complete lifetime psychiatric history, particularly for episodes occurring prior to age 18 or those managed exclusively in outpatient settings. As a result, individuals with a episode occurring >1 year prior to cohort entry or during adolescence may be misclassified as incident cases. Furthermore, our broad definition of MDD in our administrative database, likely increased sensitivity at the expense of specificity. Using a broader definition improves case ascertainment but introduces diagnostic heterogeneity. Future studies with richer diagnostic detail or validated algorithms should compare strict and broad MDD definitions.

Although administrative data provided comprehensive coverage, we could not assess medication adherence beyond dispensing records or capture over-the-counter medication use. While our relapse definition was based on medication reinitiation after a 6-month gap, which aligns with established methodologies in administrative database research, we acknowledge that medication reinitiation represents only one component of relapse. Moreover, our definition may not capture all clinically relevant relapses, particularly those managed without medication changes.

Despite adjusting for key confounders, it’s important to note residual confounding from unmeasured variables is possible. Administrative datasets lack information on disease severity, duration of untreated illness, medication adherence, substance use, lifestyle factors psychosocial support and trauma history. These factors might influence relapse trajectories and treatment decisions but were unavailable in the current data. In addition, the datasets did not allow detailed modeling of concomitant psychotropic medications (e.g., benzodiazepines, sedative-hypnotics, or adjunctive agents) or the clinical indications for their use, which may have influenced treatment patterns and reinitiation risk. Although the time-varying approach accounts for exposure changes across follow-up, it does not eliminate confounding by indication. Medication switching, augmentation, or intensification usually reflects clinical deterioration, partial response, or patient preference—all of which are not captured in administrative data. Thus, time-varying analyses may still be influenced by unmeasured clinical decision-making processes and residual bias due to severity-driven treatment changes is therefore possible.

The study’s single province setting and cohort identification through hospitalization introduce important constraints. Moreover, although our cohort was followed up in primary care and relapse was captured in primary care, our cohort was identified through hospitalization. Patients requiring hospitalization likely represent more severe cases with higher comorbidity compared to those in outpatient settings. Furthermore, patients with personality disorders, schizophrenia or bipolar disease were excluded to reduce confounding due to these conditions’ known impact on antidepressant response and poorer outcomes. Administrative data do not reliably distinguish MDD with psychotic features, single-episode psychotic depression, or prodromal psychosis. As a result, some individuals with psychotic-spectrum features may remain in the cohort, potentially contributing to heterogeneity in illness severity, hospitalization risk, and treatment switching. Given that these disorders might be common among individuals with MDD and can influence treatment outcomes, our findings may not fully represent the complex nature of MDD patients, potentially limiting the generalizability to simple MDD cases. This reinforces the need for RCTs, which remain the most rigorous approach for disentangling treatment effects from the complex interplay of comorbidities, illness severity, and unmeasured confounding.

Each treatment group likely comprises heterogeneous populations. Patients changed to combination therapy may differ in illness chronicity, treatment resistance, or comorbidity profiles, while those maintained on monotherapy may have different vulnerability patterns not observable in administrative records. Additionally, our inability to capture relapses among non-hospitalized patients, the absence of pre-hospitalization comorbidity, including chronic medical conditions such as diabetes or cardiovascular disease, medication data, and the lack of mental health symptom history before age 18 prevented comprehensive adjustment for these potential confounders. This cannot be shown in our dataset and limits interpretation of between-group comparisons.

Interpretation of the exploratory age-stratified analyses was limited by reduced statistical power and by heterogeneity within age groups. Age might overlap with unmeasured mixtures of clinical and psychosocial vulnerabilities that could influence both prescribing and relapse risk.

Despite these limitations, the study offers exploratory real-world evidence on treatment patterns and their associations with reinitiation. These observational findings should be interpreted with caution and confirmed with RCTs. The multi-faceted analytical strategy, incorporating both relative and absolute risk measures across different follow up periods and age strata offers clinically relevant insights for treatment selection in MDD in primary care. Future studies incorporating cohorts of outpatient populations would provide more comprehensive evidence disease severity in primary care.

## Conclusion

This large cohort study found that combination antidepressant therapy was associated with lower rates of reinitiation compared with SSRI monotherapy among patients hospitalized for MDD and followed in primary care. These findings should be interpreted as observational associations rather than causal effects, as treatment patterns likely reflect underlying clinical complexity, illness severity, and other unmeasured factors not captured in administrative data (e.g., early-life psychiatric history, substance use, and reasons for treatment changes). Future studies with richer clinical detail and ideally randomized designs are needed to confirm the comparative effectiveness and safety of combination strategies for relapse prevention.

## Supporting information

S1 Checklist STROBE Statement. (DOCX)

S1 Table NLCHI Databases and Study Variables. (DOCX)
