# Supplementary material for: Reinitiation of antidepressant pharmacotherapy among patients discharged from the hospital: A population-based cohort study
Source: PLOS Ment Health. 2026 Mar 25;3(3):e0000427. doi: 10.1371/journal.pmen.0000427 (PMC13016296; doi:10.1371/journal.pmen.0000427)
Supplement: S1 Table — (DOCX) [file pmen.0000427.s002.docx]

**S1 Table. NLCHI Databases and Study Variables**

| **Data Custodian** | **Name of Requested Database** | **Variable(s) Requested** | **Rationale** | **Date/Year Range of Data Requested** |
| --- | --- | --- | --- | --- |
| NLCHI | Client Registry | Year and Month of Birth | This variable is required to be able to match to patients to age.  This variable is required to provide baseline demographic of population.  This variable is required to examine differences in accessing primary care in correlation to age.  This variable is required to examine effect on incidence of outcome.  This variable is required to examine differences in treatment in correlation to age.  This variable is required as to control for confounding using high dimensional propensity scores and multivariable logistic regression. Potential confounder variables might affect the outcome and it’s important to control for these confounder variables in the  analysis. | June 2017 – Mar 2023 |
| NLCHI | Client Registry | Sex | This variable is required to be able to match to patients to age.  This variable is required to provide baseline demographic of population.  This variable is required to examine differences in accessing primary care in correlation to sex.  This variable is required to examine effect on incidence of outcome.  This variable is required to examine differences in treatment in correlation to sex.  This variable is required as to control for confounding using high dimensional propensity scores and multivariable logistic regression. Potential confounder variables might affect the outcome and it’s important to control for these confounder variables in the  analysis. | June 2017 – Mar 2023 |
| NLCHI | Client Registry | Census Sub-Division | NLCHI will review the postal code and use the PCCF+ to find the CSD. The CSD can be used independently with StatsCan Data by the researcher to gain some geographical details of the patient geographic Census Sub-Division. This information may be mapped to Health service area or paired with StatsCan publicly available income data. | June 2017 – Mar 2023 |
| NLCHI | Provincial Discharge Abstract Database  (PDAD) | De-identified Study ID for patients with a diagnosis of Depression | De-identified unique ID required for database linkage.  This variable is required as to control for confounding using univariate and multivariable conditional logistic regression analyses. Potential confounder variables might affect the outcome and it’s important to control for these confounder variables in the analysis. | June 2017 - March  2023 |
| NLCHI | PDAD | Length of Stay  (Acute vs Alternative level of care) | This variable is required for defining outcomes and for dates of outcomes.  This variable is required to control for confounding using high dimensional propensity scores and multivariable logistic regression. Potential confounder variables might affect the outcome and it’s important to control for these confounder variables in the  analysis. | June 2017 – Mar 2023 |
| NLCHI | PDAD | Date of Hospitalization/ Dischar ge | This is required to enable the team to infer prescriptions that would be prescribed after a patient hospitalization/diagnosis. Linking the prescriptions based on the hospital diagnosis will help in the analysis of the available Pharmacy network data that continue after the admission event. | June 2017 – Mar 2023 |
| NLCHI | PDAD | Episode Type  (Note on the type of depression that was present during the admission) | This variable is required for defining the outcomes. Depression episode type will give us more information on the severity of the episode, duration and treatment. This will be important for to understand our outcome, relapse of depression, better.  This variable is required as to control for confounding using univariate and multivariable conditional logistic regression analyses. Potential confounder variables might affect the outcome and it’s important to control for these confounder variables in the  analysis. | June 2017 – Mar 2023 |
| NLCHI | PDAD | Discharge Disposition | This variable is required for defining outcomes and for dates of outcomes. This variable will help us understand and define our population, severity of the disease, treatment associated with the disease and depression relapse, our outcome of interest.  This variable is required as to control for confounding using univariate and multivariable conditional logistic regression analyses. Potential confounder variables might affect the outcome and it’s important to control for these confounder variables in the  analysis. | June 2017 – Mar 2023 |
| NLCHI | PDAD | Diagnosis Code (ICD-10CA) | This variable is required for defining outcomes and for dates of outcomes. This will be very crucial in defining our study cohort, defining our population, defining our outcome and any associated comorbidities which will be associated with our secondary outcomes.  This variable is required as to control for confounding using  univariate and multivariable conditional logistic regression analyses. Potential confounder variables might affect the outcome and it’s important to control for these confounder variables in the analysis. | June 2017 – Mar 2023 |
| NLCHI | PDAD | Diagnosis Type  ICD-10CA Long Description | This variable is required for defining outcomes and for dates of outcomes.  The long description refers to the accompanying text from the PDAD. The description is just a good annotation and not a patient or record specific variable. | June 2017 – Mar 2023 |
| NLCHI | PDAD | CCI Procedure Code  (ECT and others) | This variable is required for defining outcomes and for dates of outcomes.  This variable is required to control for confounding using high dimensional propensity scores and multivariable logistic regression. Potential confounder variables might affect the outcome and it’s important to control for these confounder variables in the  analysis. | June 2017 – Mar 2023 |
| NLCHI | PDAD | CCI Long Description | This variable is required for defining outcomes and for dates of outcomes.  The long description refers to the accompanying text from the PDAD. The description is just a good annotation and not a patient or record specific variable | June 2017 – Mar 2023 |
| NLCHI | PDAD | Institution TO  Type | This variable is required for defining outcomes and for dates of outcomes.  This variable is available in PDAD, the code which identifies the level of care of the facility to which the patient was transferred for further care. This can be a good indicator that the severity of the illness requires more intensive intervention. | June 2017 – Mar 2023 |
| NLCHI | PDAD | Institution FROM Type | This variable is required for defining outcomes and for dates of outcomes.  This variable is available in PDAD, the code which identifies another health care facility or another level of care within the reporting facility from which the patient was transferred for further care. This is a good indicator of the severity and help ensure changes in care | June 2017 – Mar 2023 |
| NLCHI | MCP Physician Claims | Diagnosis Code (ICD 9, First 3 digits) | This variable is required for defining outcomes and for dates of outcomes.  This variable is required to help define depression in patients at the community level with prescriptions for treatments for depression with no hospitalizations, or hospitalization that may be outside the date range. | June 2017 – Mar 2023 |
| NLCHI | MCP Physician Claims | Service provided (Fee code) | This variable is required for defining outcomes and for dates of outcomes.  This variable is required to help define depression in patients at the community level with prescriptions for treatments for depression with no hospitalizations, or hospitalization that may be outside the date range. | June 2017 – Mar 2023 |
| NLCHI | MCP Physician Claims | Service Date | This variable is required for defining outcomes and for dates of outcomes.  This variable will be used to identify potential visits to health care provider pre and post a hospitalization for depression in the study cohort. | June 2017 – Mar 2023 |
| NLCHI | NLCHI Mortality System | Date of Death | This variable is required for defining the study exit or defining the outcome date depending on the analysis. | June 2017 – Mar 2023 |
| NLCHI | NLCHI Mortality System | Immediate Cause of Death | This variable is required for defining the study exit or defining the outcome date depending on the analysis. | June 2017 – Mar 2023 |
| NLCHI | NLCHI Mortality System | Antecedent Cause of death (all fields) | This variable is required for defining the study exit or defining the outcome date depending on the analysis. | June 2017 – Mar 2023 |
| NLCHI | NLCHI Mortality System | Conditions surrounding death (all fields) | This variable is required for defining the study exit or defining the outcome date depending on the analysis. | June 2017 – Mar 2023 |
| NLCHI | Pharmacy network | Prescription Claim Date | This variable is required to define exposure variables of interest. Our exposure is treatment prescription patterns. This will help us define our patient cohort and the duration the patients are on their prescriptions to define the outcome of interest. Our outcome is incidence of depression relapse.  This variable is required as to control for confounding using univariate and multivariable conditional logistic regression analyses. Potential confounder variables might affect the outcome and it’s important to control for these confounder variables in the  analysis. | June 2017 – Mar 2023 |
| NLCHI | Pharmacy network | Prescription Claim status | This variable is required to define exposure variables of interest. Our exposure is treatment prescription patterns. This will help us define our patient cohort and the duration the patients are on their prescriptions to define the outcome of interest. Our outcome is incidence of depression relapse.  This variable is required as to control for confounding using univariate and multivariable conditional logistic regression analyses. Potential confounder variables might affect the outcome and it’s important to control for these confounder variables in the  analysis. | June 2017 – Mar 2023 |
| NLCHI | Pharmacy network | Drug Identification Number (DIN) | This variable is required to define exposure variables of interest. Our exposure is treatment prescription patterns. This variable is important to identify specific prescription medications. This will help identify treatment prescription patterns.  This variable is required as to control for confounding using univariate and multivariable conditional logistic regression analyses. Potential confounder variables might affect the outcome and it’s important to control for these confounder variables in the  analysis. | June 2017 – Mar 2023 |
| NLCHI | Pharmacy network | Description of DIN | This variable is required to define exposure variables of interest. Our exposure is treatment prescription patterns. This variable is important to identify specific prescription medications. This will help identify treatment prescription patterns.  This variable is required as to control for confounding using univariate and multivariable conditional logistic regression analyses. Potential confounder variables might affect the outcome and it’s important to control for these confounder variables in the  analysis. | June 2017 – Mar 2023 |
| NLCHI | Pharmacy network | DIN Dosage | This variable is required to define exposure variables of interest. Our exposure is treatment prescription patterns. This variable is important to identify specific prescription medications. This will help identify treatment prescription patterns. This variable is required as to control for confounding using univariate and multivariable conditional logistic regression analyses. Potential confounder variables might affect the outcome and it’s important  to control for these confounder variables in the analysis. | June 2017 – Mar 2023 |
| NLCHI | Pharmacy network | AHFS therapeutic class code | This variable is required to define exposure variables of interest. Our exposure is treatment prescription patterns. This variable is important to identify specific prescription medications This will help identify treatment prescription patterns.  This variable is required as to control for confounding using univariate and multivariable conditional logistic regression analyses. Potential confounder variables might affect the outcome and it’s important to control for these confounder variables in the  analysis. | June 2017 – Mar 2023 |
| NLCHI | Pharmacy network | ATC code (All Levels) | This variable is required to define exposure variables of interest. Our exposure is treatment prescription patterns. This variable is important to identify specific prescription medications. This will help identify treatment prescription patterns.  This variable is required as to control for confounding using univariate and multivariable conditional logistic regression analyses. Potential confounder variables might affect the outcome and it’s important to control for these confounder variables in the  analysis. | June 2017 – Mar 2023 |
| NLCHI | Pharmacy network | Drug Brand Name | This variable is required to define exposure variables of interest. Our exposure is treatment prescription patterns. This variable is important to identify specific prescription medications. This will help identify treatment prescription patterns. Moreover, this will help with the descriptives of prescription data.  This variable is required as to control for confounding using univariate and multivariable conditional logistic regression analyses. Potential confounder variables might affect the outcome and it’s important to control for these confounder variables in the  analysis. | June 2017 – Mar 2023 |
| NLCHI | Pharmacy network | Drug Generic Name | This variable is required to define exposure variables of interest. Our exposure is treatment prescription patterns. This variable is important to identify specific prescription medications. This will help identify treatment prescription patterns. Moreover, this will help with the descriptives of prescription data.  This variable is required as to control for confounding using univariate and multivariable conditional logistic regression analyses. Potential confounder variables might affect the outcome and it’s important to control for these confounder variables in the  analysis. | June 2017 – Mar 2023 |
| NLCHI | Pharmacy network | DIN label name | This variable is required to define exposure variables of interest. Our exposure is treatment prescription patterns. This variable is important to identify specific prescription medications. This will help identify treatment prescription patterns. Moreover, this will help with the descriptives of prescription data.  This variable is required as to control for confounding using univariate and multivariable conditional logistic regression analyses. Potential confounder variables might affect the outcome and it’s important to control for these confounder variables in the  analysis. | June 2017 – Mar 2023 |
| NLCHI | Pharmacy network | Drug Strength | This variable is required to define exposure variables of interest. Our exposure is treatment prescription patterns. This variable is important to identify specific prescription medications. This will help identify treatment prescription patterns. This variable is required as to control for confounding using univariate and multivariable conditional logistic regression analyses. Potential confounder variables might affect the outcome and it’s important  to control for these confounder variables in the analysis. | June 2017 – Mar 2023 |
| NLCHI | Pharmacy network | Quantity Dispensed | This variable is required to define exposure variables of interest. Our exposure is treatment prescription patterns. This variable is important to identify specific prescription medications. This will help identify treatment prescription patterns.  This variable is required as to control for confounding using univariate and multivariable conditional logistic regression analyses. Potential confounder variables might affect the outcome and it’s important to control for these confounder variables in the  analysis. | June 2017 – Mar 2023 |
| NLCHI | Pharmacy network | Dispensed Days Supply | This variable is required to define exposure variables of interest. Our exposure is treatment prescription patterns. This variable is important to identify specific prescription medications. This will help identify treatment prescription patterns. This will help with the descriptives of prescription data. This variable will also help with identifying the outcome of interest. Our outcome is incidence of depression relapse.  This variable is required as to control for confounding using univariate and multivariable conditional logistic regression analyses. Potential confounder variables might affect the outcome and it’s important to control for these confounder variables in the  analysis. | June 2017 – Mar 2023 |
